# Evaluation of Vascular Endothelial Growth Factor A and Selected Parameters of Coagulation and Fibrinolysis in a Group of Patients with Subarachnoid Haemorrhage

**DOI:** 10.1155/2019/8759231

**Published:** 2019-07-08

**Authors:** Michał Wiciński, Abu Sitta Al Drawi, Bartosz Malinowski, Wioleta Stolarek

**Affiliations:** ^1^Department of Pharmacology and Therapeutics, Faculty of Medicine, Collegium Medicum in Bydgoszcz, Nicolaus Copernicus University, 9 Sklodowskiej-Curie Street, Bydgoszcz, Poland; ^2^Department of Neurosurgery and Neurotraumatology, University Hospital No. 2 in Bydgoszcz, Collegium Medicum in Bydgoszcz, Nicolaus Copernicus University, 75 Biziel Street, Bydgoszcz, Poland

## Abstract

*Introduction. *Subarachnoid hemorrhage (SAH) is currently one of the most serious diseases of the central nervous system. To reduce the negative consequences of SAH and help clinicians to assess the patient's condition, there are attempts to search for new diagnostic markers, which quickly and accurately allow for the proper diagnosis. The aim of this research was the concentration and activity of Vascular Endothelial Growth Factor A (VEGF-A) and selected parameters of coagulation and fibrinolysis in the blood of patients with SAH. Serum levels of VEGF-A in patients diagnosed with SAH are measured to assess the correlation between VEGF-A and the clinical condition of patient. This may help with proper therapeutics and better prognosis.* Methods. *The study involved 85 patients with subarachnoid hemorrhage. The control group consisted of 45 healthy subjects, sex and age matched. The following parameters were determined: APTT (Activated Partial Thromboplastin Time), INR (International Normalized Ratio), D-dimers and fibrinogen concentration, and the concentration of VEGF-A by ELISA (R&D USA).* Results. *The average concentration of VEGF-A in the study group was significantly lower compared to the control group. The D-dimer concentration was higher in patients with SAH but the difference was not significant. Coagulation parameters such as INR, APTT, and fibrinogen did not show significant differences between investigated groups.* Conclusions. *VEGF-A cannot be an independent marker of SAH. Selected parameters of coagulation and fibrinolysis such as D-dimers, INR, APTT, and fibrinogen should not be used as markers of SAH.

## 1. Introduction

Nowadays subarachnoid hemorrhage (SAH) is a major cause of death and permanent disability of patients. Despite significant progress in the field of medical imaging of the central nervous system SAH is still a major challenge for clinicians [1]. Expanding knowledge of the pathogenesis of SAH contributes to more effective prevention and makes it possible to minimalize the side effects and improve the prognosis [1, 2]. Subarachnoid hemorrhage is defined as extravasation of blood from a vessel to a subarachnoid space. SAH can be idiopathic, but is more often caused by injuries and vascular malformations. The highest percentage of nontraumatic subarachnoid hemorrhage being up to 85% comes from the bursting aneurysms forming on the cerebral blood vessels [3]. The SAH incidents caused by rupture of aneurysms are influenced by patients' ethnicity and a place of living. One of the highest incidences of SAH in Europe is recorded currently in Finland being up to 23 patients per 100000 inhabitants, whereas in China it is only 2 patients per 100000 population [4, 5]. In Poland SAH caused by the rupture of the aneurysm is observed in 10 of 100 000 patients per year. Death is found in over 25% of patients [1, 6]. Diagnostic difficulties and uncertain prognosis of patients with subarachnoid hemorrhage contribute to the search for new biomarkers which could facilitate diagnosis and rapid assessment of patients' biomarker recovery [3, 7]. The ideal biomarker should be characterized by a high sensitivity, specificity, and simplicity of the assay method. Furthermore its level should correlate with cerebrospinal fluid concentration. The new marker, being characterized by high prognostic and predictive value, would definitely facilitate therapeutic procedures [8–10]. One of the main problems of research focused on looking for the new markers, as far as SAH is concerned, is their lack of specificity [11]. Therefore, the determination of the concentration of only one biochemical marker is not sufficient in laboratory diagnostics [12]. The sensitivity and specificity of the biomarkers analyzed separately are low, but they have increased when combined [13]. Serum levels of Vascular Endothelial Growth Factor A (VEGF-A) in patients diagnosed with SAH are measured to assess the correlation between VEGF-A and the clinical condition of patient [14, 15]. The assessment of coagulation system and fibrinolysis parameters during the SAH shows the activation of these systems and it is sufficient in the evaluation of thromboembolic complications. Some of these markers belong also to an acute phase proteins [16].

### 1.1. Vascular Endothelial Growth Factor A (VEGF-A)

VEGF is a mitogenic cytokine which consists of six isoforms designated by the letters from A to E. VEGF family of proteins take part in the initiation of a cellular response by receptor tyrosine kinases. So far two receptors specific for VEGF: VEGFR-1 (Flt1) and VEGFR-2 (KDR/Flt-1), have been identified. Most effects at the cellular level result from interaction of VEGFR-2 receptor with its ligand VEGF-A [17, 18]. VEGF-A is produced by T lymphocytes, macrophages, and platelets. The factors which have a decisive influence on the stimulation of the synthesis of this cytokine are hypoxia, hypoglycemia, and interleukin-1 (IL-1) and interleukin-6 (IL-6) [4]. One of the important physiological functions of VEGF-A is its “participation” in the regulation of angiogenesis and proliferation of lymphatic vessels. At the same time an increasingly important role is attributed to VEGF-A in terms of its function as the increasing vascular permeability factor, as well as an important element in the process of vascular endothelial growth [18, 19]. Various studies concerning the processes of angiogenesis and factors involved in it have become the key to the origins of disease entities evoked by different reasons including, for instance, hypoxia and ischemia of the brain [4, 17, 20]. Some authors also analyzed the correlation between the concentration of VEGF-A and the advancement of cancer or conditions involving angiogenesis in the tissues. Serum level VEGF-A in patients diagnosed with SAH is measured to assess the correlation between VEGF-A and the clinical condition of a particular patient. This may help with proper therapeutics and better prognosis [14, 15].

### 1.2. The Selected Parameters of Coagulation and Fibrinolysis in Patients with SAH

Activated Partial Thromboplastin Time (APTT) is used as a general screening test for the detection of intrinsic coagulation disorders. APTT is sensitive to the absence or deficiency of factors VIII, IX, XI, XII, X, and II, and prekallikrein, high molecular weight kininogen (HMWK), and fibrinogen [21]. Prolonged APTT is often associated with an increased risk of bleeding; hence it requires some explanation. Most often the invalid value of this ratio is observed in patients with hemophilia and von Willebrand disease. Massive extravasation of blood, both intraoperatively and spontaneously, is also reflected in the APTT. In most cases its value is extended [22]. International Normalized Ratio (INR) is a standardized ratio of prothrombin time. It reflects the efficiency of the extrinsic coagulation. It comprises, among others, the factors II, V, VII, and X [1]. Fibrinogen and factors II, V, and X are produced in the liver; at the same time they are dependent on vitamin K and its deficiency causes prolongation of prothrombin time. In everyday medical practice INR is mainly used to control an oral anticoagulant therapy. The normalized ratio of prothrombin time-INR was used in order to assess the coagulation system in patients with SAH [23–25]. D-dimers as products of fibrin degradation are released into the circulation during fibrinolysis; elevations of D-dimers have been associated with ischemic stroke and SAH; it is also a prognostic factor for stroke progression [26].

## 2. Methods

The study was approved by the Bioethics Committee of the Ludwik Rydygier Collegium Medicum in Bydgoszcz, the Nicolaus Copernicus University in Toruń. The study involved 85 patients with subarachnoid hemorrhage aged from 29 to 81 including 47 women and 38 men. Patients were hospitalized at the Department of Neurosurgery and Neurotraumatology, University Hospital No. 2 in Bydgoszcz. The control group consisted of 45 healthy subjects, sex and age matched. Blood samples were collected from the cubital vein puncture with minimal venous stasis into tubes containing 3.2% sodium citrate up to 24 hours after subarachnoid hemorrhage. In derogation of time no longer than 2 hours after sampling, samples were centrifugated and the resulting plasma was analyzed. The following parameters were determined: APTT, D-dimers, prothrombin time, fibrinogen concentration, and the concentration of VEGF-A by ELISA (R&D USA). The study was performed after positive approval by the Bioethics Commission.

### 2.1. Statistical Analysis

Statistical analysis was conducted by using a software program STATISTICA 7.1 for Windows from StatStoft company. The variability of quantitative parameters was described by using the positional-average median (Me) and quartiles: lower (Q1) and upper (Q2). Testing normality of variables was performed with the Shapiro-Wilk test and showed that it had nonnormal distribution. Therefore, the significance of differences between groups was tested by nonparametric Mann-Whitney U test. Statistically significant differences were accepted at* p <0.05.*

## 3. **Results**

The average concentration of VEGF-A in the study group was significantly lower and was 43.39 ± SD compared to the control group in which the average concentration was 63.31± SD (*p=0.0008*) (Table 1). Higher concentrations of VEGF-A were found in male patients; however, the obtained values were not significantly different (Table 1). We did not observe the relationship between the concentration of VEGF-A and the age of the patients (Table 1). The health status of the patients was estimated using the Hunt-Hess scale and the scale of Fischer. In the group of 85 patients suffering from subarachnoid hemorrhage 66 (75%) were classified in the I-III ° and 19 (25%) in IV ° Hunt-Hess scale. As far as the Fischer scale is concerned, 52 (61%) patients were classified in I-II °, whereas 33 patients (39%) were classified in the III-IV °. There were no differences in the concentration of VEGF-A in patients examined by both scales (Table 2). The concentration of fibrinogen (2.8 g/L) in patients who had SAH was slightly lower compared to the control group (2.8 ± SD vs 3.43± SD, respectively) (*p=0.1529*) (Table 3). The D-dimer concentration was higher in patients with SAH but the difference was not significant. In other parameters of coagulation (INR, APTT) there were no statistically significant differences between the study groups.

## 4. Discussion

Diagnostic difficulties and subsequent treatment of patients with subarachnoid hemorrhage lead clinicians to searching for new markers to facilitate a treatment procedure. This study results in determining the concentration of VEGF-A in serum of patients suffering from SAH. The decrease in the concentration of VEGF-A concerning the treatment group is significant, but it is not a specific marker as far as subarachnoid hemorrhage is concerned. This is implied by no difference in the concentration between the groups of patients whose evaluation was based on the Hunt and Hess scale and the Fisher scale.

SAH directly leads to vasoconstriction and hypoxia of brain cells [20, 27]. In a study conducted by Jośko it was proved that hypoxia of cells results in the expression of VEGF-A in rats [15, 28]. The results of this study show that the SAH incident leads to increased angiogenesis in the cerebral hemispheres and cerebellum and the increased concentration of VEGF-A.

The Eicker study demonstrated that the expression of VEGF-A is increased during cerebral ischemia when it comes to people and animals. In this study, by analysing the cerebrovascular microperfusion, vasospasm induction was observed immediately after the SAH incident. The increase in the concentration of VEGF-A was also observed, which is associated with neoangiogenesis increase in cerebral vessels [29].

During Drevs' and Scheufler's experiment it was proved that the complex morphological and biochemical changes within the microvasculature of the brain after SAH are related to, among others, the expression of molecules such as VEGF-A, tumor necrosis factor (TNF), and fibroblast growth factor [30]. At the same time the correlation between performed surgery in patients with SAH and the increase in the concentration of VEGF-A was observed. Probably it has to do with the accumulation of platelets around the ventricles of the brain and cerebrospinal fluid.

SAH activates the coagulation and fibrinolysis system in the early phase of the disease. In the Peltonen et al. study D-dimers and fibrinogen levels were higher in patients with SAH, while APTT was shortened [31]. In addition, a more severe clinical condition was associated with a higher concentration of D-dimers.

Fujii et al. demonstrated that the activation of the coagulation system and fibrinolysis, measured by an increase of D-dimers, occur when blood reaches the subarachnoid space. Hematopoietic blood cells without SAH do not have such a large impact on the systemic activation of the coagulation system, despite local activation in the tissue directly surrounding the hematoma of the brain. This confirms the hypothesis that the presence of blood in the subarachnoid space is responsible for the systemic activation of the coagulation system [32].

Suzuki et al. showed that the activation of fibrinolysis in the cerebrospinal fluid is the highest between 3 and 5th day after surgery and decreases between 12th and 14th days, whereas in the blood it is the opposite. The level of D-dimers is significantly reduced in the group of patients with cerebral vasospasm [33].

The increase in fibrinogen concentration, which occurs under the influence of various reasons, is one of the elements of the acute phase reaction resulting from the interaction of many cytokines, mainly interleukins 1 and 6 [34]. Peltonen et al. found an increase in fibrinogen concentration in SAH, but also on the first and seventh day after surgery [31].

Ettinger pointed out that increased levels of fibrinogen in the blood may correlate with worse treatment results [35]. Maurice-Williams and Wanatabe et al. observed an increased level of fibrinogen products in the blood and cerebrospinal fluid, in patients with symptoms of cerebral vasospasm and distant ischemic changes in the brain [36, 37].

Many factors influence the clinical course of SAH. Neuroimaging studies remain the basis of diagnosis. However, an additional study of biochemical markers is an important direction in laboratory diagnostics.

## 5. Conclusion

(1) VEGF-A cannot be an independent marker of SAH.

(2) Selected parameters of coagulation and fibrinolysis such as D-dimers, INR, APTT, and fibrinogen should not be used as markers of SAH.

## Figures and Tables

**Table 1:** Comparison of VEGF-A concentrations in SAH patients and control group. Comparison of the concentrations of VEGF-A, depending on the gender, on the age and the prevalence of diabetes of patients with subarachnoid haemorrhage.

Parameter	*Patients with SAH (n=85)*		*Control group (n=45)*		
	Me	Q1; Q3	Me	Q1; Q3	p
VEGF-A [ng/mL]	43,39	30,71	63,31	54,59	0,0008
				71,39	
		67,48			

Parameter	*Men (n=38)*		*Women (n=47)*		
	Me	Q1; Q3	Me	Q1; Q3	p

VEGF-A [ng/mL]	46,74	31,11	40,78	27,14	ns
		67,74		65,88	

Parameter	*Age < 50 (n=34)*		*Age > 50 (n=51)*		
	Me	Q1; Q3	Me	Q1; Q3	p

VEGF-A [ng/mL]	42,64	30,71	43,39	27,80	ns
		71,02		65,88	

**Table 2:** Comparison of the concentrations of VEGF-A by the Hunt and Hess scale and by the Fisher scale.

Parameter	The Hunt and Hess scale				p
	I°-III° (n=66)		IV° (n=19)		
	Me	Q1; Q3	Me	Q1; Q3	
VEGF-A	40,66	25,40	44,27	36,40	ns
[ng/mL]		67,59		57,27	

Parameter	The Fisher scale				p
	I°-II° (n=52)		III°-IV° (n=33)		
	Me	Q1; Q3	Me	Q1; Q3	

VEGF-A	40,66	27,14	44,27	32,40	ns
[ng/mL]		72,77		52,95	

**Table 3:** Comparison of selected parameters of coagulation and fibrinolysis in the study and the control group.

Parameter	Group	Value of the parameter	p
		(mean ± SD)	
Fibrinogen [g/L]	SAH patients	2,8±1,7	ns
	Control	3,4±2,6	

INR	SAH patients	0,97±1,72	ns
	Control	0,92±0,84	

APTT [s]	SAH patients	29,0±4,0	ns
	Control	32,0±5,0	

D-dimer [mg/mL]	SAH patients	431,2±218,7	ns
	Control	166,8±187,6	

## Data Availability

The data used to support the findings of this study are available from the corresponding author upon request.
